# Recommencement of anti-thrombotics following traumatic intracranial haemorrhage; a preliminary survey of UK consultant neurosurgeons informing the RESTART-tICrH trial design

**DOI:** 10.1016/j.bas.2026.106667

**Published:** 2026-09-06

**Authors:** Jack Read, Peter Whitfield, Michael D. Jenkinson, Catherine J. McMahon, Ellie Edlmann

**Affiliations:** a University of Exeter Medical School, Exeter, United Kingdom; b Peninsula Medical School, Faculty of Health, University of Plymouth, Plymouth, United Kingdom; c Department of Neurosurgery, University Hospitals Plymouth NHS Trust, Plymouth, United Kingdom; d Department of Neurosurgery, The Walton Centre NHS Foundation Trust, Liverpool, United Kingdom; e Institute of Systems, Molecular and Integrative Biology, University of Liverpool, Liverpool, United Kingdom; f Centre for Clinical Neurosciences, Northern Care Alliance, Salford Royal, Manchester, United Kingdom

**Keywords:** Antithrombotics, Aging, Elderly, Head injury

## Abstract

**Introduction:**

Traumatic intracranial haemorrhage (tICH) commonly follows falls in elderly people, around half of whom are taking anti-thrombotic (AT) medications pre-injury. ATs are typically stopped acutely, but guidance on safe recommencement is limited.

**Research question:**

We surveyed UK neurosurgical consultants to understand variations in AT recommencement practice and inform trial design.

**Materials and methods:**

An online cross-sectional survey was distributed via the Society of British Neurological Surgeons mailing list to assess self-reported consultant practice regarding cessation and recommencement of AT therapy following tICH.

**Results:**

Sixty-two consultant neurosurgeons responded. Following tICH most are very likely or likely to stop AT therapy for AF (92%), stroke prevention (85-90%), or previous MI (78%). There was greater variation when managing those with cardiac stents, with only 59% stopping ATs. Recommencement typically occurred at two weeks post-injury in both conservatively (50%) and surgically (40%) managed patients, with ∼80% of neurosurgeons restarting ATs within four weeks. Most defined early AT resumption to be one-week (72%), while late resumption was more varied at four (39%) and six (31%) weeks. Willingness to randomise was highest in lower-risk cases. More caution was shown to randomising patients with cardiac indications for AT therapy, greater injury severity, and reduced consciousness.

**Discussion and conclusion:**

These findings highlight the need for better evidence and guidance in this patient group. Based on the survey findings, a trial design targeting patients on AT therapy for AF or secondary VTE prevention, randomised to early (<7 days) versus late (4 weeks) resumption appears a relevant trial question.

## Highlights

• Wide variation exists in UK practice for restarting anti-thrombotics after traumatic intracranial haemorrhage (tICH).
• Most neurosurgeons stop anti-thrombotics for AF, stroke, or MI after tICH.
• Anti-thrombotics are typically restarted between 2 and 4 weeks post-injury.
• Early AT resumption commonly defined as <1 week; late as 4–6 weeks.
• Survey supports feasibility of trial comparing early vs late AT restart.

## 1 Introduction

Traumatic intracranial haemorrhage (tICH) is a common consequence of injury in the elderly following low-energy falls (Edlmann et al., 2023; Peeters et al., 2015; de Wit et al., 2021). Older adults are at increased risk of tICH due to cerebral atrophy, increased likelihood of falling, and use of anti-thrombotic (AT) medications (de Wit et al., 2021; Büchele et al., 2020; Batchelor and Grayson, 2012). AT medication, including antiplatelets and oral anticoagulants (OAC), are commonly prescribed in older adults to reduce thromboembolic risk in atrial fibrillation (AF), prior cerebrovascular events (CVE), and cardiovascular disease (Hart et al., 2007; Nishimura et al., 2014). Pre-injury use of anti-thrombotic (AT) medications is reported in around 50% of older patients presenting with head injury, increasing the risk of tICH and worsening outcomes (Edlmann et al., 2023; Scotti et al., 2020).

The cessation of AT therapy following tICH is standard practice in order to minimise risk of haemorrhagic expansion (Edlmann et al., 2023). However, prolonged interruption exposes patients to thromboembolic complications, including ischaemic stroke and cardiovascular events, which may cause additional morbidity or death (Edlmann et al., 2023; Hart et al., 2007; Nishimura et al., 2014). The decision to restart AT therapy must balance two competing risks: early recommencement, which may predispose to intracranial re-bleeding or expansion of the initial injury, versus delayed recommencement, which increases the duration of thromboembolic vulnerability (Edlmann et al., 2023). The literature suggests that re-bleeding rates are relatively low at around 4% in the first 30 days, whereas the rate of thromboembolism increases over time from around 4% in the first week to 10% by week four (Milling et al., 2021; Connolly et al., 2019). The balance of risks and benefits is complicated by individual factors such as injury severity and underlying thromboembolic risk factors (de Wit et al., 2021; Büchele et al., 2020; Hart et al., 2007; Nishimura et al., 2014).

In the absence of evidence-based guidelines to direct clinical practice, clinical decisions are often made on a case-by-case basis, driven by clinician judgement and local practice rather than standardised national protocols (Edlmann et al., 2023). The aim of this preliminary study was to explore self-reported practice and perspectives among UK consultant neurosurgeons regarding the timing of AT recommencement following tICH, to identify areas of consensus and variation, and to inform on future clinical trial design randomising between “early” and “late” AT resumption.

## 2 Methods

### 2.1 Study design

A preliminary cross-sectional survey formed part of the development work for the RESTART-tICrH trial. It was designed to examine self-reported practice and perspectives among UK consultant neurosurgeons regarding cessation and recommencement of AT therapy following tICH and identify variations in clinical decision-making. The survey was designed by neurosurgeons with a specialist interest in TBI and anti-thrombotics, in consultation with cardiologists and elderly care physicians. It was not intended to characterise multidisciplinary practice but rather to establish neurosurgical opinions in common patient groups referred on ATs to inform on future trial development.

### 2.2 Participants and recruitment

Consultant neurosurgeons in the UK were invited to participate. The survey invitation was distributed through the Society of British Neurological Surgeons (SBNS) mailing lists, and local neurosurgical departments, with recipients encouraged to share with colleagues more widely. The number of eligible consultants who received the invitation and extent of onward distribution were not recorded, consequently a formal response rate could not be calculated. The survey was open from October 2020 to December 2020.

### 2.3 Survey

The questionnaire consisted of six sections, designed to capture key aspects of AT management in patients with tICH (Supplementary Material 1). The sections covered:

1. Decision making around stopping AT therapy in clinical context
2. Timing of AT recommencement
3. Role of repeat imaging prior to recommencement of AT therapy
4. The definition of “early” versus “late” recommencement of AT therapy
5. Willingness to randomise “early” versus “late” recommencement of AT therapy in specific clinical scenarios
6. Patient group suitability for clinical trial

### 2.4 Data collection & analysis

An online survey platform (surveymonkey) was used to collect and store survey data. Descriptive statistics were used to summarise responses, including proportions and frequency distributions for categorical variables.

### 2.5 Ethical statement

Formal ethical approval was not required as the study involved a survey of consultant practice without direct patient involvement. Participation was voluntary, and no individual personal details were collected.

## 3 Results

At the time of survey, there were 453 consultant neurosurgeons recorded as practicing in the UK and Ireland (Whitehouse et al., 2020) and 62 survey responses were received.

### 3.1 Cessation of anti-thrombotic therapy

**Fig. 1:**
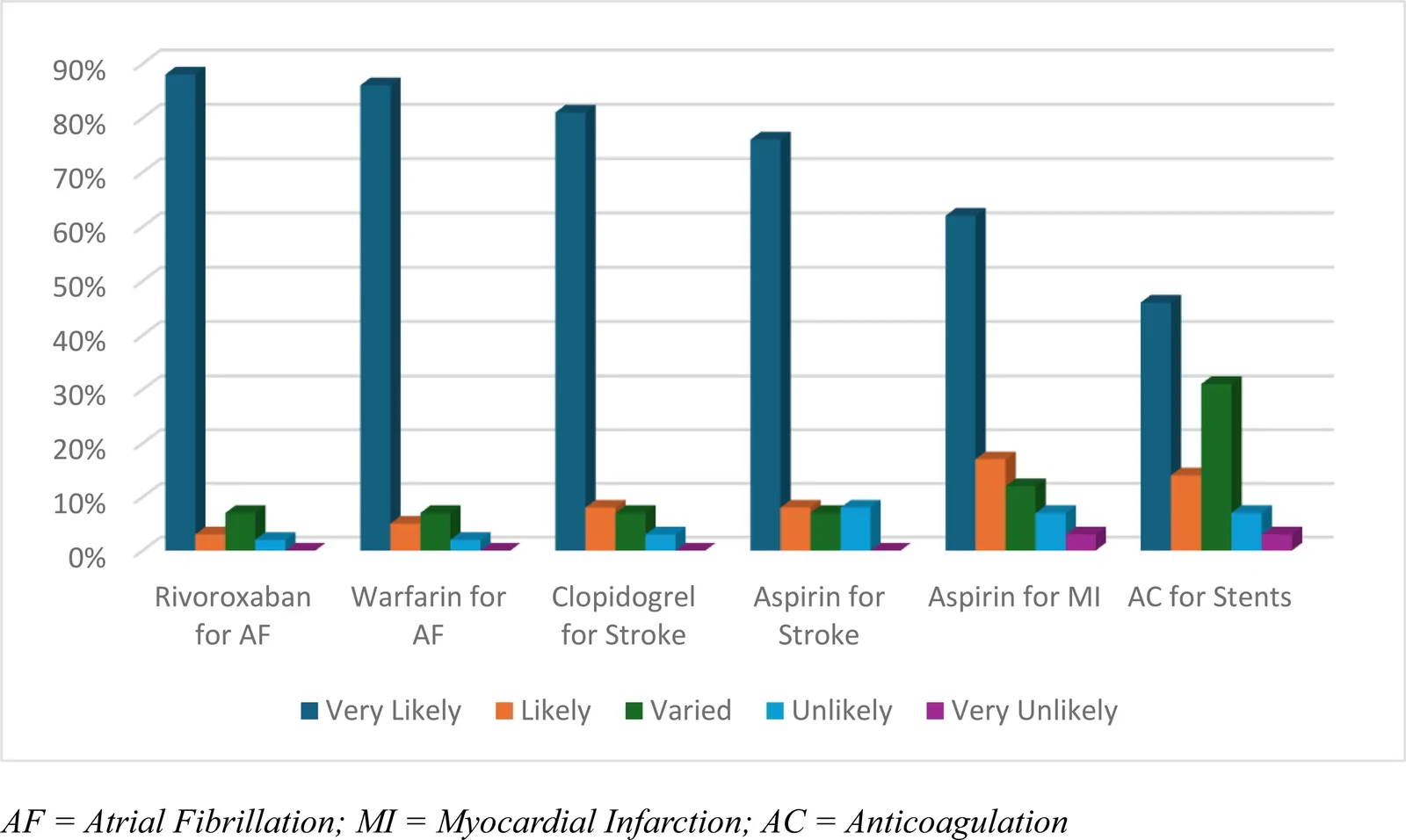
Likelihood of consultants advising cessation of AT therapy in older adults with tICH AF = Atrial Fibrillation; MI = Myocardial Infarction; AC = Anticoagulation.

Most consultants were very likely or likely to advise cessation of AT therapy in older adults with tICH when concerning anti-coagulation for AF (54/59, 92%) and anti-platelets for stroke prevention (50-53/59, 85%-90%) or previous MI (47/60, 78%). For antiplatelet therapy in cardiac stents, only 59% (35/59) would stop, while 31% (18/59) reported a varied approach and 10% (6/59) were unlikely to discontinue therapy in this group (see Fig. 1).

### 3.2 Timing of anti-thrombotic recommencement

**Fig. 2:**
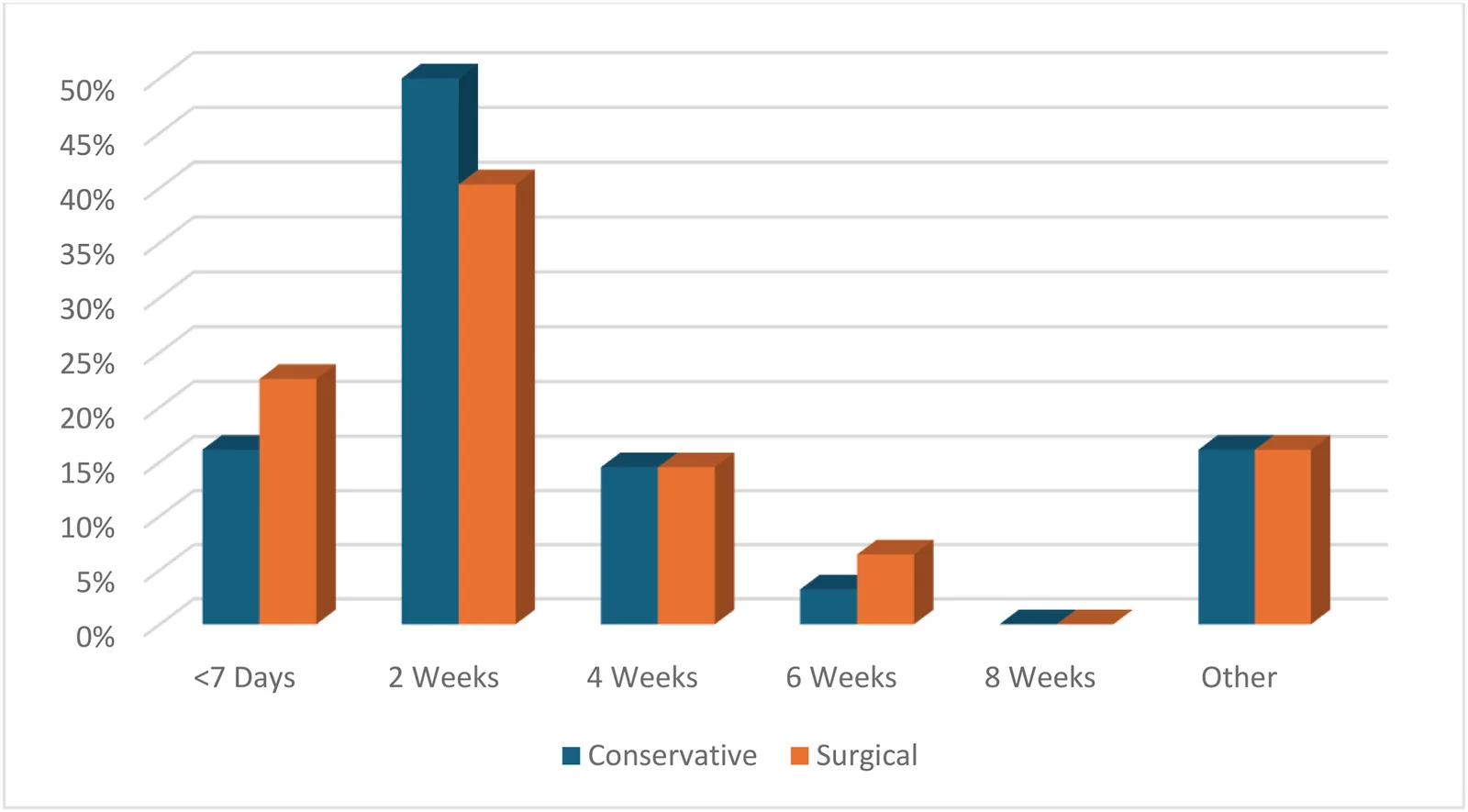
Optimal timing of recommencement of anti-thrombotics.

The most common timing for recommencement of ATs was at two weeks post-injury in both conservatively (31/62, 50%) and surgically (25/62, 40%) managed tICH (Fig. 2). A total of 80% (50/62) of neurosurgeons reported restarting them within four weeks, with 84% (52/62) at 6 weeks. A free text option was available for “other” (15% respondents) with most of these comments relating to a need for a tailored approach depending on patient factors and indication. Some consultants reported waiting 12 weeks to recommence and others that the AT is permanently stopped in patients who were considered high risk of falls or injury.

### 3.3 Use of repeat imaging prior to recommencement

Only 11% (7/62) of surgeons *always* request a follow-up CT scan prior to resuming AT therapy, while 24% (15/62) *usually* did, and a further 40% (25/62) only *sometimes* did. The remainder reported using imaging *rarely* (13/62, 21%) or *never* (2/62, 3%) prior to AT recommencement.

### 3.4 Defining “early” vs “late” resumption

**Fig. 3:**
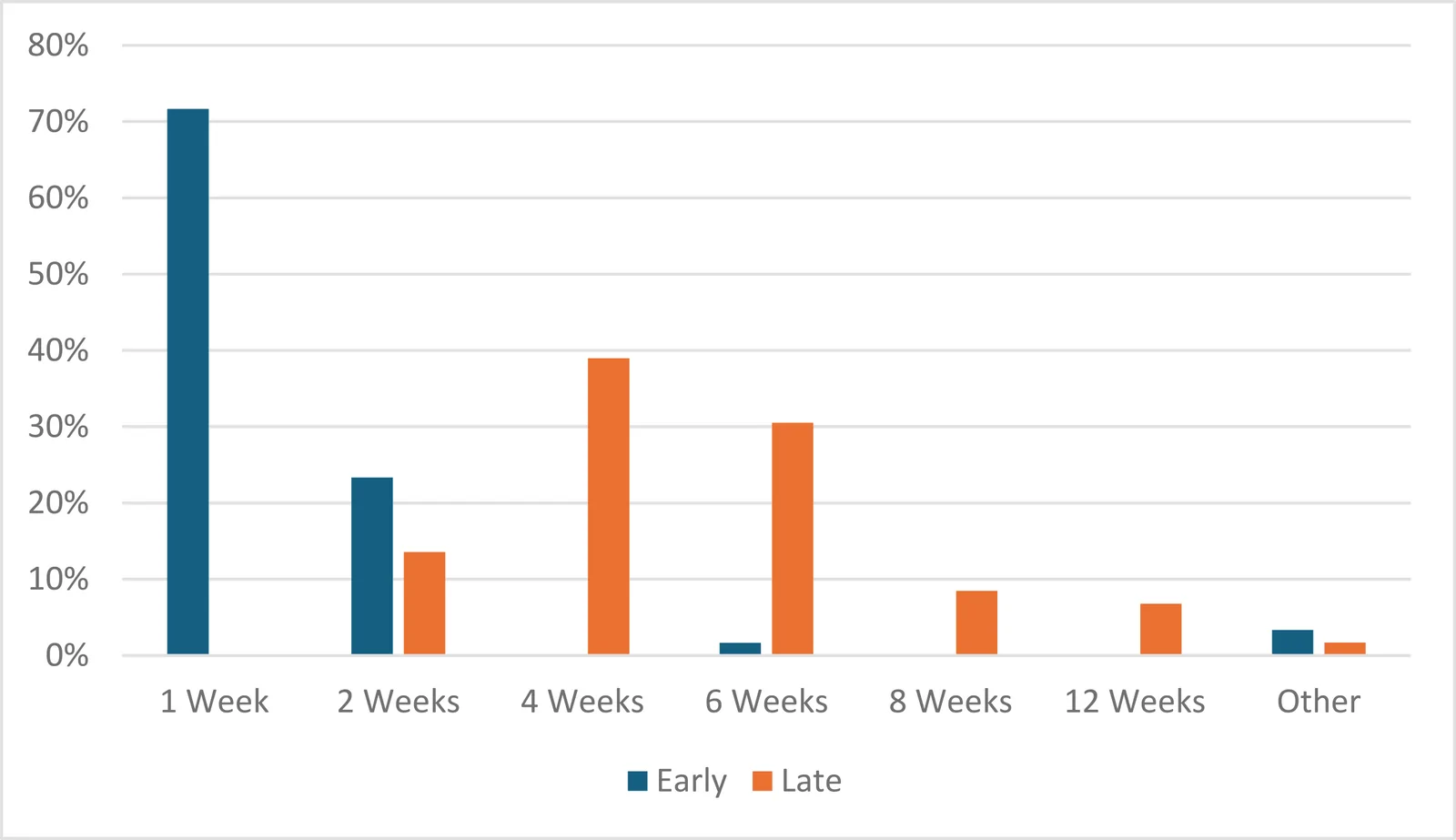
Defining “Early” vs “Late” Resumption.

Neurosurgeons demonstrated varied interpretations of the definition for “early” and “late” resumption of ATs (Fig. 3). Early resumption was most commonly considered to occur at one week (43/60, 72%) post injury. Late resumption was most frequently defined as four weeks (23/59, 39%) or six weeks (18/59, 31%).

### 3.5 Willingness to randomise

**Fig. 4:**
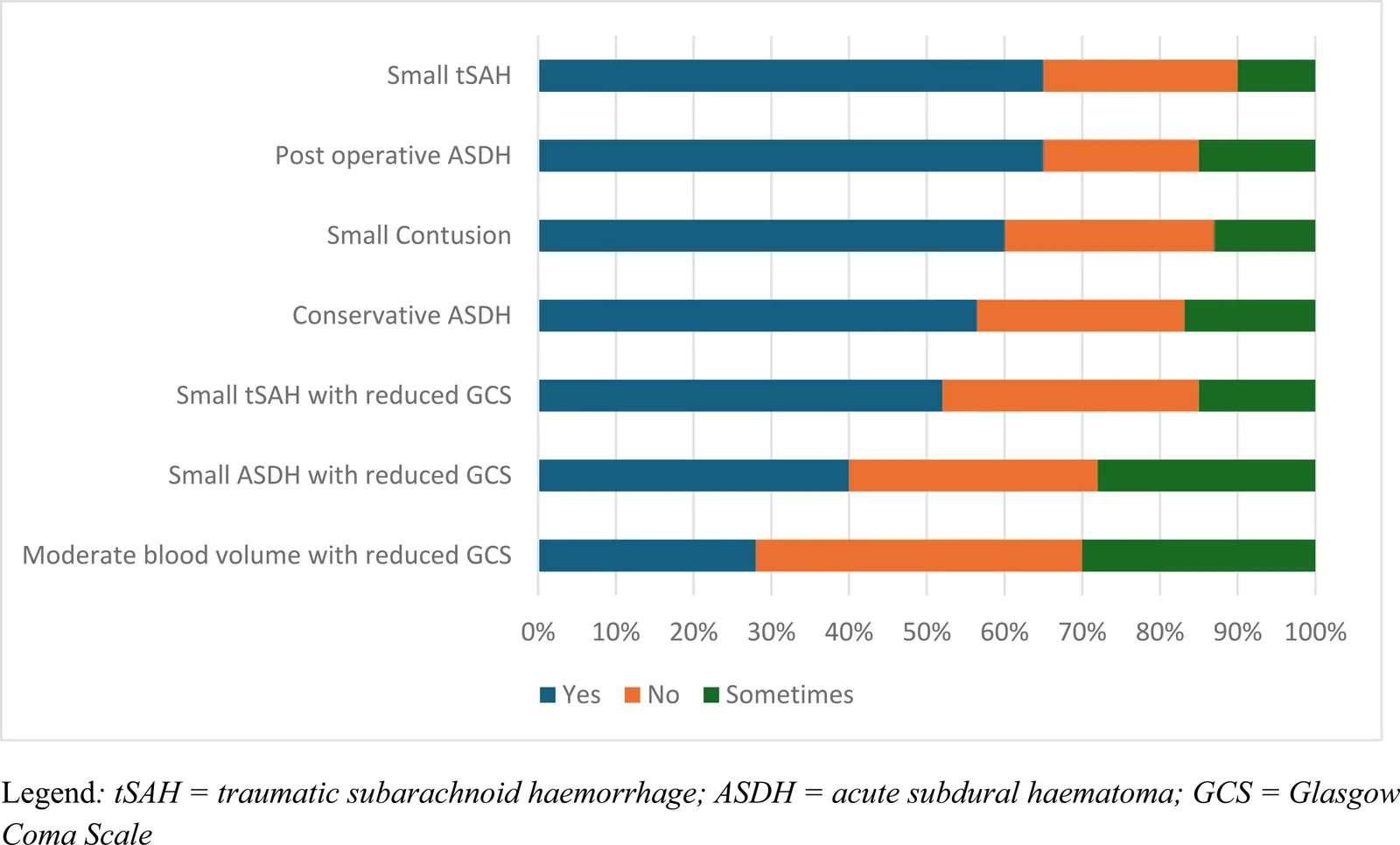
Willingness to randomise in a trial Legend*: tSAH = traumatic subarachnoid haemorrhage; ASDH = acute subdural haematoma; GCS = Glasgow Coma Scale*.

Willingness to randomise to “early” versus “late” AT therapy recommencement varied by clinical scenario (Fig. 4). The greatest willingness was seen in cases of small-to-moderate volume tSAH managed conservatively (39/60, 65%), post-operative ASDH (39/60, 65%), cerebral contusions (36/60, 60%) and conservatively managed ASDH with a normal GCS (34/60, 57%). A lower willingness to randomise was reported in conservatively managed patients with GCS reduced from baseline (GCS 12-14) for all pathologies; small-volume tSAH (31/60, 52%), small volume ASDH (24/60, 40%), and moderate-volume ASDH or contusions (17/60, 28%). However, even in these patient groups, an average of a further 24% said they would sometimes randomise.

### 3.6 Patient group suitability for clinical trial

**Fig. 5:**
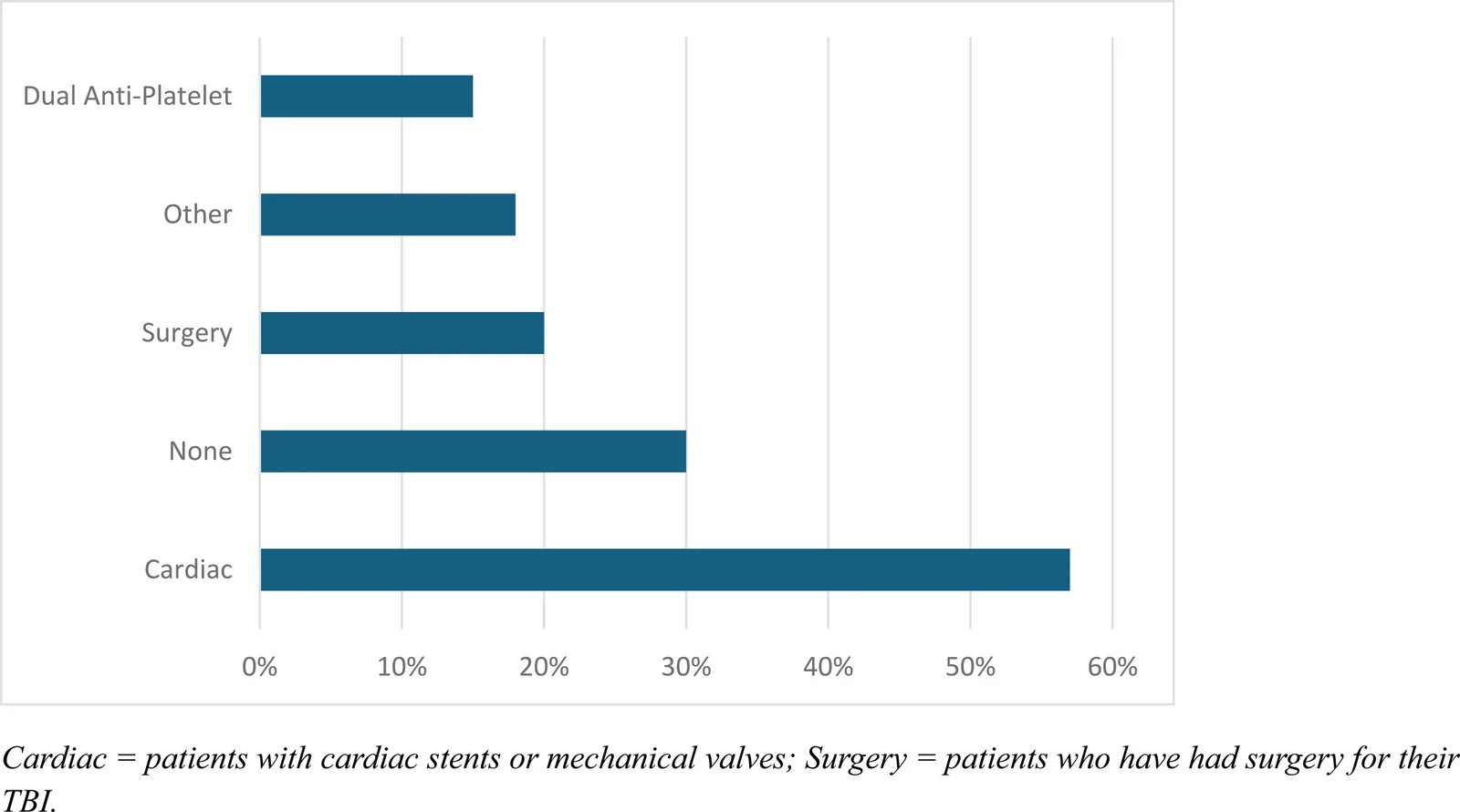
Patient Exclusion from a trial Cardiac = patients with cardiac stents or mechanical valves; Surgery = patients who have had surgery for their TBI.

Most respondents (34/60, 57%) would not be willing to enrol patients with cardiac stents or valves in a trial (Fig. 5). Less than 20% would exclude post-operative patients (12/60, 20%) or those on dual antiplatelets (9/60, 15%).

## 4 Discussion

Managing AT therapy following tICH poses several issues, central to which is the need to balance the risks of haemorrhagic progression against thromboembolic events, both of which can result in major disability or death (Edlmann et al., 2023; Scotti et al., 2020; HAWRYLUK et al., 2010). This preliminary national survey provides an insight into self-reported neurosurgical practice and opinions among respondents, critical to clinical trial design. It revealed both areas of relative consensus but with variability in clinical decision-making which is not surprising given the limited evidence base and lack of clinical guidelines. This survey was intended to inform on neurosurgical practice, and therefore cannot inform on what other multi-disciplinary teams might advise in similar situations (e.g. cardiologists or stroke physicians). However, in the authors experience, it is often left to the neurosurgeons to take the lead on guiding in decision making around ATs after head injury despite the broad and complex indications for ATs and associated individual patient risk factors. The primary aim of this work was to help determine whether there was a specific patient cohort which might be suitable for a clinical trial where equipoise exists but in which neurosurgeons would be willing to randomise.

Our clinical practice survey found that temporary cessation of AT therapy is common in the acute phase of tICH, particularly in patients on treatment for AF or for secondary prevention of stroke & MI. This finding is in consistent with the current literature, where concern around early rebleeding often outweighs the risk of thromboembolism in the immediate post-injury period (Edlmann et al., 2023; HAWRYLUK et al., 2010; AlKherayf et al., 2017; Maeda et al., 2012; Hawryluk et al., 2011). The approach to managing patients on AT therapy for high-risk cardiac indications, such as coronary stents or mechanical heart valves, remains an area of variability. Only 59% of respondents routinely discontinued AT therapy in this group, while a further 31% indicated a variable approach. This likely reflects concerns around the thromboembolic consequences of treatment interruption in this population, which even for a short period, can lead to stent and valve thrombosis, and is associated with increased mortality (Eisenberg et al., 2009; Kinlay et al., 2021; Cannegieter et al., 1994; Leiria et al., 2011). It is not feasible, or appropriate, to incorporate all the indications for AT within a single trial on recommencement timing, and it is more challenging to harmonise treatment algorithms in higher risk populations. Given this, and the results of the survey indicating the highest consensus (>90%) of stopping oral anti-coagulation in patients with AF this appears a promising patient cohort for a clinical trial. As AF is one of the most prevalent and growing indications for oral anti-coagulation in our aging population, with a prevalence around 6% in adults over 65 growing to 22% in those over 80, it would be large population to target with a potential for significant impact on clinical practice (Khurshid et al., 2023; Linz et al., 2024; Bo et al., 2024).

The timing of AT resumption is a key area of uncertainty. Our findings show a preference for restarting therapy at two, four or six-weeks following injury. This suggests that clinicians view this timeframe as a window in which the risk of thromboembolic events starts to progressively outweigh the risk of rebleeding. While the existing literature remains inconclusive on the exact point that this happens, we do know that early rebleeding is relatively low at around 4% and that thrombosis risk increases to 10% by four weeks (Milling et al., 2021; Connolly et al., 2019). Limited high-quality prospective trauma specific data exists and current evidence is largely made up from spontaneous ICH (sICH) studies (Edlmann et al., 2023; AlKherayf et al., 2017; Maeda et al., 2012; Hawryluk et al., 2011; Al-Shahi Salman et al., 2019). From the sICH literature we know that it is safe to restart ATs after a haemorrhage, and that the risk of thrombotic events outweighs the risks of rebleeding in the long-term, but there is not a clear consensus on the optimal timepoint to restart ATs in this group (Al-Shahi Salman et al., 2019; Balali et al., 2023; Best et al., 2022; Murthy et al., 2017). Due to the differing aetiology and risk factors associated with tICH these findings may not be directly applicable, further outlining the necessity for a trial in this cohort.

A recent systematic review on tICH patients on ATs reported a 25% rate of new or progressive haemorrhage on routine imaging performed within 12-72 h of injury which justifies the need to stop these medications (Edlmann et al., 2023). However, fewer than 10% were clinically significant highlighting the discrepancy between radiological progression and clinical deterioration, raising questions about whether prolonged withholding of AT medication is always necessary (Edlmann et al., 2023). Late progressive haemorrhage is rarely reported but only six of 59 studies reported rates or timing of recommencement of ATs. Furthermore, the majority of included OAC studies related to patients on warfarin, despite direct oral anticoagulants (DOACs) now being the first line anticoagulant for AF (Edlmann et al., 2023; Bo et al., 2024). Converse to the haemorrhage risk, the risk of thrombotic complications is known to increase over time in the absence of AT therapy (Edlmann et al., 2023; Hart et al., 2007; Nishimura et al., 2014). In the sICH population, withholding AT medications is associated with increased long-term thromboembolism risk, suggesting early recommencement may be beneficial in some patients (Al-Shahi Salman et al., 2019; Murthy et al., 2017). Some limited evidence in the tICH systematic review suggested that early resumption of ATs, within 7-14 days, may not only be safe but could be beneficial. However, a large, randomised trial is still needed to provide robust evidence for safe recommencement timing in the tICH population (Edlmann et al., 2023).

In this survey, only 11% of respondents routinely require a follow-up CT scan prior to recommencement of AT therapy, with most using imaging selectively. The literature remains divided on this issue, some support routine imaging to detect haemorrhagic progression, arguing that it may influence outcomes (Bee et al., 2009). Others argue that radiologic progression, without clinical deterioration, is of little clinical value and advocate for symptom guided repeat imaging (Washington and Grubb, 2012; Huang et al., 2020). In relation to pragmatic clinical trial design, there is a lack of clear benefit for routine repeat imaging and necessitating this within a trial is only likely to increase the burden of imaging in future clinical practice.

When considering how to determine the best time point for recommencing ATs through a randomised clinical trial, it is not feasible to test a wide spectrum of timing and therefore within the survey we sought to assess what neurosurgeons would consider “early” and “late” timing. Whilst there was some variability in the survey respondents, a clear majority considered one week to represent “early” resumption, and this aligns with the earliest time point considered safe in the literature (Edlmann et al., 2023). The definition of “late” resumption was less consistent with 39% defining it as four weeks and 31% as six weeks. This variability in “late” resumption reflects a broader inconsistency in the literature. The definition of “late” resumption in sICH studies often depends on the indication for AT therapy, patients with mechanical heart valves having late resumption defined as early as one week (Barra et al., 2024), and those with AF or stroke often having 4 weeks as the definition of late resumption (Liu et al., 2023). The absence of similar guidance in tICH leaves clinicians to rely on individual judgement, resulting in variable practice as seen in this survey. When considering the balance of risks in VTE with prolonged cessation of AT, for the purposes of a trial a “late” resumption time point of 4 weeks is likely to minimise increased VTE risk and was the preferred option of the two later points in the survey.

This survey did not address the issue of prophylactic low-molecular-weight-heparin (LMWH), which is often used for thromboprophylaxis in admitted tICH patients. This was felt to be a separate question, which incorporates all tICH patients (not just those previously on anti-thrombotics), and is specific to in-patient thrombotic risk. This is being addressed in the TOP-TBI trial (ISRCTN64696029) (ISRCTN64696029, 2024). Regardless of the use of prophylactic LMWH, the time point for safe therapeutic anticoagulation still needs to be determined. In-patient stay for milder tICH in the older population may also be very short, with a drive for earlier discharge, many of these patients could be managed at home and therefore will need clear instruction on their medication timing (Santing et al., 2025).

Designing interventional trials in this population is challenging due to the heterogeneity of patients with tICH. Our findings identify key areas that provide preliminary information relevant to trial design including a focus on oral anticoagulants in patients with AF, evidence of clear variability and thus equipoise in the timing of recommencement but reasonable agreement on dichotomised time points for early and late resumption. Respondents were more comfortable randomising lower-risk patients, with smaller-volume haemorrhages, whereas caution was expressed in cases involving reduced consciousness or higher thromboembolic risk. This is unsurprising and reflects the wide range of severity of tICH and thus the variation in perceived risk of new and evolving haemorrhage over time. This exemplifies the need to incorporate clinician discretion in trial design and not being too strict on eligibility criteria to reflect real-world practice where patient cases and risk factors vary. It also further supports the need to focus on one clinical population (patients on oral anticoagulation for AF) rather than potentially more complex patients, and the majority of respondents felt that patients with cardiac stents or valves would not be suitable for a trial.

The data from this survey informed further clinical trial development, alongside broader stake-holder engagement (cardiology, stroke, elderly care physicians and emergency department physicians), patient and public perspectives and clinical trials methodologists. This survey data provides some baseline information to help us understand changes in opinion and practice over time, as informed by clinical trials results, such that we can understand the impact of such trials in the longer term.

### 4.1 Limitations

This study has several limitations. As a survey-based study, the findings report self-reported practices, which may not represent real-world behaviour. Recall bias and subjective interpretation of the clinical scenarios may have influenced responses and the survey questions were not validated prior to circulation. Although the survey was distributed via the SBNS mailing list, the possibility of selection bias cannot be excluded, the exact number of consultants who received the survey invitation and the extent of forward distribution were not recorded. Respondents represented a self-selected group, and no non-responder analysis was possible, therefor the views captured may not fully represent all UK consultant neurosurgeons. None-the-less as data in the literature is scare in this area, and national audit difficult and time-consuming, we are reliant on the “best available” data from engaged individuals surveyed to support development of clinical research questions.

The survey intentionally focussed on neurosurgical perspectives and therefore does not provide a comprehensive assessment of multidisciplinary practice. While the survey included a broad range of clinical scenarios, treatment indications, and antithrombotic therapies, it did not capture all indication, treatment, or pathology specific factors that may influence recommencement decisions. The aim was to identify a common patient population which may be suited to a trial.

## 5 Conclusion

This pragmatic survey of UK consultant neurosurgeons highlights variability among the respondents in the management of AT therapy following tICH. In the absence of clear evidence-based guidelines, clinicians are required to make difficult decisions regarding AT therapy cessation and recommencement based on a case-by-case basis. These findings highlight the need for better quality data and an indication of consensus that this could be achieved in a pragmatically designed trial. Alongside the best available evidence and wider stakeholder consultation, the findings contributed to the development of the RESTART-tICrH trial, which is now recruiting patients receiving AT therapy for AF or secondary prevention of VTE, randomising to early (1 week) versus late (4 weeks) resumption (Santing et al., 2025; Clinical Trials Research Centre. RESTART tlCrH [Internet]).

## Ethics declaration

Written informed consent to take part in the study and to publish the article has been obtained from all participants or their legal representatives. The privacy rights of participants have been observed.

This study was performed in compliance with relevant laws, regulatory frameworks and guidelines where the research took place. Ethics committee approval was not required under relevant laws and institutional guidelines. Formal ethical approval was not required as the study involved a survey of consultant practice without direct patient involvement. Participation was voluntary, and no individual personal details were collected.

## Disclosures

The authors have no personal, financial, or institutional interests to declare.

## Declaration of competing interests

The authors declare that they have no known competing financial interests or personal relationships that could have appeared to influence the work reported in this paper.
