# Supplementary material for: Recommencement of anti-thrombotics following traumatic intracranial haemorrhage; a preliminary survey of UK consultant neurosurgeons informing the RESTART-tICrH trial design
Source: Brain Spine. 2026 Sep 6;6:106667. doi: 10.1016/j.bas.2026.106667 (PMC13578408; doi:10.1016/j.bas.2026.106667)
Supplement: Supplementary file 1 [file mmc1.docx]

**Supplementary material: Questionnaire**

1. For older patients presenting with traumatic haemorrhage on CT (e.g. tSAH, ASDH or contusion) how likely are you to advise STOPPING the anti-thrombotic in the following indications; very likely, likely, varied, unlikely, very unlikely
i. Aspirin for primary stroke prevention
ii. Aspirin for previous MI
iii. Warfarin for AF
iv. Rivaroxaban for AF
v. Clopidogrel from stroke prevention
vi. Aspirin/clopidogrel for cardiac stent
2. On average, when would you normally advise re-starting anti-thrombotics if stopped?
i. <7 days
ii. 2 weeks
iii. 4 weeks
iv. 6 weeks
v. 8 week
vi. Other
3. Would you normally advise repeat CT before re-starting anti-thrombotics?
i. Always
ii. Usually
iii. Sometimes
iv. Rarely
v. Never
4. If a patient was randomised to "early" or "late" resumption of anti-thrombotics when would you consider these time points to be? (can give multiple answers)
i. 1 week
ii. 2 weeks
iii. 4 weeks
iv. 6 weeks
v. 8 weeks
vi. 12 weeks
vii. Other
5. Please consider the following clinical examples in older patients (>65) and whether you would be willing to randomise to early (e.g 2-4wks) or late (e.g. 8 weeks) resumption of the anti-thrombotics. **Yes/No/In Some Cases**
i. Normal baseline GCS and small/medium volume traumatic subarachnoid (tSAH) on CT managed conservatively
ii. Normal baseline GCS and acute subdural haematoma (ASDH) managed conservatively
iii. Normal baseline GCS with small/medium cerebral contusions managed conservatively
iv. GCS 12-14 (reduced compared to baseline) with small volume tSAH managed conservatively
v. GCS 12-14 (reduced compared to baseline) with small ASDH managed conservatively
vi. GCS 12-14 (reduced compared to baseline) and moderate blood load (contusion or ASDH) managed conservatively
vii. Normal baseline GCS post-operatively following evacuation of ASDH
6. Please tick any patient groups you would exclude from such a trial
i. Patients who require surgery (even if randomised post-operatively)
ii. Patients taking anti-thrombotics for cardiac indications (e.g. metallic valve/stent)
iii. Patients on dual anti-platelets
iv. None specific
v. Other (please specify)
